# Transcriptome and Metabolome Analyses Reveal the Involvement of Multiple Pathways in Flowering Intensity in Mango

**DOI:** 10.3389/fpls.2022.933923

**Published:** 2022-07-14

**Authors:** Qingzhi Liang, Kanghua Song, Mingsheng Lu, Tao Dai, Jie Yang, Jiaxin Wan, Li Li, Jingjing Chen, Rulin Zhan, Songbiao Wang

**Affiliations:** ^1^Key Laboratory of Tropical Fruit Biology, Ministry of Agriculture, South Subtropical Crops Research Institute, Chinese Academy of Tropical Agricultural Sciences, Zhanjiang, China; ^2^College of Tropical Crops, Yunnan Agricultural University, Puer, China; ^3^Zhanjiang Experimental Station, Chinese Academy of Tropical Agricultural Sciences, Zhanjiang, China; ^4^College of Agriculture, Guangxi University, Nanning, China; ^5^Haikou Experimental Station, Chinese Academy of Tropical Agricultural Sciences, Haikou, China

**Keywords:** FLOWERING LOCUS, flowering mechanism, inflorescence, light quality pathway, photoperiod pathway, reproductive phase, vernalization pathway

## Abstract

Mango (*Mangifera indica* L.) is famous for its sweet flavor and aroma. China is one of the major mango-producing countries. Mango is known for variations in flowering intensity that impacts fruit yield and farmers' profitability. In the present study, transcriptome and metabolome analyses of three cultivars with different flowering intensities were performed to preliminarily elucidate their regulatory mechanisms. The transcriptome profiling identified 36,242 genes. The major observation was the differential expression patterns of 334 flowering-related genes among the three mango varieties. The metabolome profiling detected 1,023 metabolites that were grouped into 11 compound classes. Our results show that the interplay of the FLOWERING LOCUS T and CONSTANS together with their upstream/downstream regulators/repressors modulate flowering robustness. We found that both gibberellins and auxins are associated with the flowering intensities of studied mango varieties. Finally, we discuss the roles of sugar biosynthesis and ambient temperature pathways in mango flowering. Overall, this study presents multiple pathways that can be manipulated in mango trees regarding flowering robustness.

## Introduction

Mango (*Mangifera indica* L.) fruit is famous for its flavor, sweetness, and aroma. Major mango-producing countries include India, China, Thailand, Indonesia, and Pakistan. Although mango is economically significant, it is commonly known for its variations in flowering, which has a major impact on the fruit yield and profitability (Mukherjee, 1953). When fruit trees (such as avocado, mandarin, lychee, and mango) are cultivated in subtropical regions, they show alternate bearing. It gives a very good yield 1 year and poor yield during the following year (Muñoz-Fambuena et al., 2011). Therefore, a lack of uniformity in fruit yield is considered highly undesirable from a breeding point of view because it results in severe and unexpected economic losses (Goldschmidt, 2013). It is difficult to comprehend such behavior of mango due to some natural traits including high heterozygosity, long juvenile phase, substantial fruit drop, and the requirement of larger areas for a conclusive evaluation (Lal et al., 2017). However, the availability of various modern facilities of genomics and molecular characterization is facilitating the understanding of flowering behavior not only in model plants but also in field and fruit crops (Sharma et al., 2019, 2020).

Flowering is a vital developmental stage in the life cycle of plants. The floral transition is mediated through an interplay of specific climatic conditions (light and temperature variations) and genetic factors. The use of classical and molecular genetic approaches has identified four major flowering pathways in Arabidopsis and other plants (Kim, 2020): the vernalization pathway, the gibberellin (GA) pathway, the autonomous pathway, and the photoperiod pathway. Additionally, more pathways have been reported in this context, which include ambient temperature-, hormones-, and sugar-dependent pathways (Rolland et al., 2002; Seo et al., 2011; Moghaddam et al., 2013; Wahl et al., 2013; Conti, 2017; Susila et al., 2018). However, further research is expected to elucidate the comprehensive role of these pathways in flowering. Earlier studies have generally reported the response of flowering genes to diverse environmental and/or physiological stimuli (Yoo et al., 2010; Halder and Abu Hasan, 2020; Patil et al., 2021). Several factors were found to be associated with irregular flowering. These include, but are not limited to, phytohormones-mediated intracellular signaling and regulatory pathways (Ionescu et al., 2016), metabolism of macromolecules (Shalom et al., 2012), nutritional factors (Yanik et al., 2013), crop load (Shalom et al., 2014), modulation of flower induction-related genes (Guitton et al., 2016), and transcription factors (Kwak et al., 2016). In mango, vegetative growth relies on reserves of carbohydrates that also affect the fruit-bearing pattern (Goldschmidt, 2013). Advancements in omics are proving to be helpful in understanding the molecular mechanisms of flowering switches (Turktas et al., 2013). The famous FLOWERING LOCUS T (FT)-like and gibberellins metabolism genes were isolated by Nakagawa et al. (2012) from biennially bearing trees of mango. Moreover, elevated expression of the LFY, AP1, and FT genes was reported in mango leaves at the time of flower induction. Contemporary advancements in molecular genetics of flowering in plants have identified novel aspects of floral stimulus (Bao et al., 2020; Kim, 2020; Finnegan et al., 2021). The FT gene encodes a protein that is localized in the vascular veins (phloem tissue) of leaves. The FT gene is activated by CONSTANS (CO) gene-encoded protein, and its protein product serves as the florigenic component buds (Corbesier et al., 2007). This inference is reinforced by the translocation of *Hd3a*-encoded protein (an ortholog of FT in rice) from leaves to buds. The Hd3a protein apparently represents the florigen in this plant (Tamaki et al., 2007). Moreover, *PtFT1*-encoded protein (an ortholog of aspen) and CONSTANS regulate growth cessation and flowering time of *Populus trichocarpa* (Böhlenius et al., 2006). Once the FT protein is translocated to buds, it interacts with FD (a bZIP transcription factor) to trigger the expression of genes related to floral identity (like APETALA1 or AP1) (Wigge et al., 2005). Analogous mechanisms are potentially active in mango. However, the dynamics of gene expression can be significantly altered. For a better understanding of flowering initiation, differential gene expression analysis among divergent genotypes is expected to provide meaningful insights (Shalom et al., 2014). Moreover, previous studies have focused either on the transcriptome (Sharma et al., 2020) or the metabolome of mango for flowering or other traits (Tan et al., 2018, 2020; Shivashankara et al., 2019).

In this context, the present study was conducted to understand the expression profiling of flowering-related genes of different pathways. Three types of mango varieties differing in flowering intensity, i.e., easy-to-flower type, intermediate type, and hard-to-flower type, were selected for the current study. We explored the genetic and metabolic differences between easy-to-flower varieties and difficult-to-flower varieties, leading to an increase in our understanding of the genetic control of the flowering in the varieties differing in flowering intensity.

## Materials and Methods

### Plant Material

**Figure 1:**
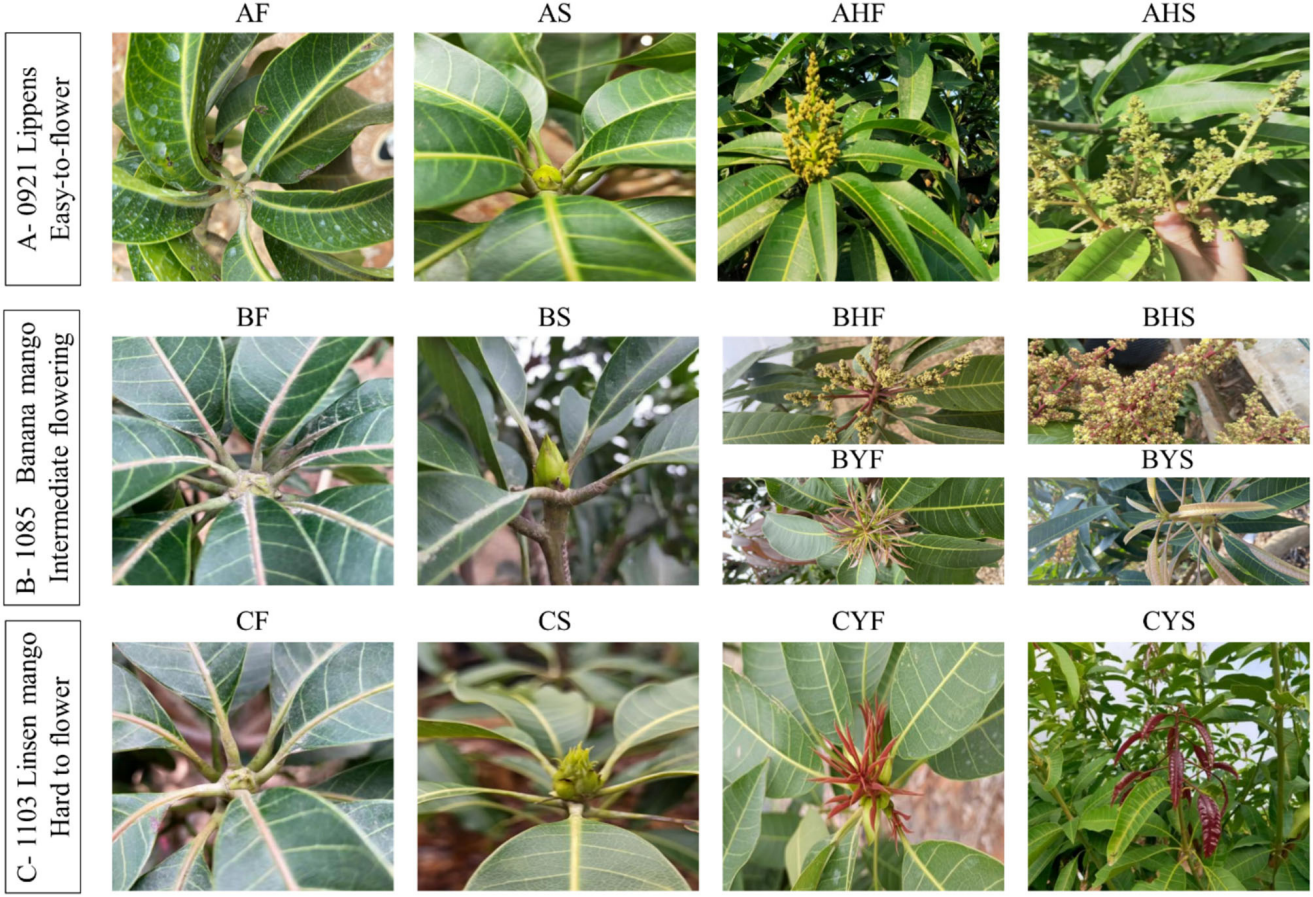
A representation of stages of flowering in mango. The samples were collected from distinct stages for the analysis of transcriptome and metabolome.

Three mango varieties differing in flowering intensity were included in the study. The first variety is Lippens (0921) (A) (Grajal Martín et al., 2009), which flowers easily and normally. The second variety is Banana mango (1085) (B). The Banana mango trees are of two types, i.e., the first type includes the trees that bear flowers, while the second type does not bear flowers. The third variety is Linsen mango (1103) (C); the trees of this type of mango are considered hard to flower. Linsen mango is an old landrace in the Hainan province (since 1970's) and has not been widely promoted in production due to difficulties in flowering. It was introduced as a germplasm resource from the Hainan Province to National Field Genebank for Tropical Fruit (Zhanjiang, China) in 2002; however, so far, it has never flowered and borne fruit despite being 19 years old. The mango trees are growing in National Field Genebank for Tropical Fruit (Zhanjiang, China, 21°9'N, 110°16'E). The ages of the trees are 12, 13, and 19, years, respectively. The mean temperature, humidity, and day length during the flowering time of the selected varieties are 26~29°C, 65~73%, and 11:56', respectively. The soil type is the latosolic red soil. The field management followed conventional standard field practices. The sampled tissues from the varieties A, B, and C were meristem, flower bud, and inflorescences in phase I (mid-size panicle early anthesis stage) and phase II (full-size panicle maximum anthesis stage). For variety B, two additional tissues were collected, i.e., leaf bud 3 and leaf bud 4, for the trees that bear no flowers. The sample naming strategy is explained in Table 1 and Figure 1.

**Table 1:** Details of the studied tissues of three mango varieties.

**Variety**	**Tissue details**			
	**Meristem**	**Flower bud**	**Inflorescence phase I**	**Inflorescence phase II**
Lippens (0921) A	AF	AS	AHF	AHS
	Dormant	Flower bud differentiation	0921	0921 flower 4
Banana Mango (1085) B	BF	BS	BHF	BHS
	Dormant	Flower bud differentiation	Flower 3	Flower 4
			BYF	BYS
			Leaf bud 3	Leaf bud 4
Linsen Mango (1103) C	CF	CS	CYF	CYS
	Dormant	Flower bud differentiation	Leaf bud 3	Leaf bud 4

### Analysis of Transcriptome

#### Extraction of RNA and Sequencing

We extracted the total RNA from 14 tissues of mango varieties in three replicates. For this purpose, the Spin Column Plant Total RNA Purification kit (Sangon Biotech Co., Ltd., China) was used. After extraction, RNA integrity was tested by agarose gel electrophoresis, purity was checked by NanoPhotometer, and the concentration was measured on a spectrophotometer and Qubit 2.0 Fluorometer. Once the quality was confirmed, the mRNAs were obtained using polyA tail enrichment of RNAs through Oligo (dT) magnetic beads. The mRNAs were fragmented by adding fragmentation buffer, followed by cDNA synthesis using a cDNA synthesis kit (QuantiTech Reverse Transcription Kit, Qiagen). Later on, AMPure XP beads were used to purify cDNA. After purification, the cDNA was A-tailed and ligated with sequencing adapters. The fragment size selection was done using AMPure XP beads followed by obtaining cDNA libraries. The Qubit 2.0 was used for quantification of the library, and the detection of insert size was performed by Agilent 2000. A qRT-PCR was used for the quantification of the effective library concentration, i.e., >2 nM. After finding out the effective library concentration, libraries were pooled and sequenced on the Illumina HiSeq platform.

#### Sequencing Data Analyses

First of all, the sequencing data were filtered in order to remove reads with adapters, having N content >10%, and paired-end reads. This resulted in obtaining high-quality reads. Sequencing error rate distribution and GC content distribution were checked as reported earlier (Chen et al., 2019). HISAT2 was used to compare transcriptome sequencing data with the reference genome (https://www.ncbi.nlm.nih.gov/assembly/GCA_016746415.1) and the comparison efficiency was summarized as a Microsoft Excel ® table.

BLAST was used to compare the transcript sequences with the KEGG (Kanehisa, 2000), Swiss-Prot (Apweiler et al., 2004), and GO (Ashburner et al., 2000) databases. The transcript expression was represented as Fragments Per Kilobase of transcript per Million fragments mapped (FPKM), and the overall distribution of the gene expression was represented as a box plot. An R suit (ropls) was used for principal component analysis (PCA) and hierarchical cluster analysis, and PCC between the samples was computed and represented as heatmaps in R using the pheatmap and cor functions.

We used DESeq2 to determine the gene expression variations among the tissues of the same mango variety or among different mango varieties (Love et al., 2014). To find the false discovery rate (FDR), multiple hypothesis test correction was applied to the hypothesis test probability (*p*-value) as described by the Benjamini–Hochberg method. The screening criteria for the differentially expressed genes (DEGs) were |log2 Fold Change| ≥1 and FDR <0.05. Venn diagrams were prepared in InteractiVenn (Heberle et al., 2015). The KEGG (https://www.genome.jp/kegg) was used for pathway annotation of the DEGs (Kanehisa, 2000). The enrichment of DEGs in different KEGG pathways was done as reported earlier (Chen et al., 2019). The enrichment results were represented as scatter charts.

#### qRT-PCR Analysis

The qRT-PCR-based expression analysis was performed for 19 genes. The Primer-BLAST tool (https://www.ncbi.nlm.nih.gov/tools/primer-blast/) was used for primer designing (Table 2). An internal control gene (*Actin*) expression was used for normalization. The qPCR and data analysis were performed as reported earlier (Livak and Schmittgen, 2001). The reactions were performed in triplicate, and means were used to calculate the expression using 2^−ΔΔct^ method.

**Table 2:** List of primers used for qRT-PCR analysis.

**Gene ID**	**Forward primer**	**Reverse primer**	**Gene description**
*Mi00g06850.1*	TGGTCCCTCCTATCATTAC	GCATTCCTCTTGCGATTT	GIGANTEA
*Mi00g13610.1*	GCGACCACGACATCCACT	CCTACTGACATCACCACCTCC	Zinc finger CONSTANS
*Mi01g06850.1*	TAGTAGACCCTCTTGTTGTG	CAGACTTGCCTGTTATTGT	TERMINAL FLOWER 1
*Mi02g06210.1*	GGCAAATTCTGGTAAGC	AATCATCTCCCATCACATC	Histone deacetylase HDT1-like
*Mi02g15810.1*	TGTGGTGGTACAGGTGAG	GAGAAATCTATTGGCTTGA	TERMINAL FLOWER 2
*Mi02g20180.1*	TTGTTGTTGGGCGAGTT	CTTCCACCAATAGAAACCC	FLOWERING LOCUS T/HEADING DATE 3A
*Mi03g23990.1*	GAAGGTGAAAGCCAAAGAG	CGGAGCCAACCACAAGC	Serine/threonine-kinase WNK1-like
*Mi04g17530.1*	CATCGAGGCAGAGTCAAG	TCATTAAAGGTCCCAAGC	TEMPRANILLO 1
*Mi04g18420.1*	TCTTTGTGATGCCGATGT	GAGACCAGTTACCCGTTG	APETALA1-like
*Mi05g09970.1*	GAGTGCGAGCCTACATTG	TCATGGCAACCATCCTG	Agamous-like MADS-box AGL9 homolog
*Mi05g22170.1*	CGGAGGTTGCCCTTATC	TCTTCAGTTGGCTGCTTA	MADS-box SOC1
*Mi07g04370.1*	TTAAGAAGGAGGATGTCAACT	TTTATCACCCAAACCAAGC	Serine/threonine-kinase WNK1-like
*Mi08g02850.1*	TAACAAGCCTGAAACGG	AACGCTACACGAATCCA	SOC1-like 2
*Mi08g02930.1*	GCTAACCAGCCTGAAAC	ACTACACGAACCCAAATC	SOC1-like 2
*Mi04g17530.1*	GCGTGTTCCGATTCTGG	CCTCATCTTCATCTCCCTCC	Zinc finger CONSTANS-LIKE 2-like
*Mi09g01410.1*	AAAACCCAAATGAGACGC	AAGATGATAAGGGCAACC	MADS-box SOC1
*Mi10g11120.1*	GGTTAGTTCAGGCATTGGT	CTCTGAGGCAACTCTGGTAT	FLOWERING LOCUS D
*Mi12g02330.1*	AAGACGATTCGGTATCATTCA	GGTCAACTTCGGATTCCAC	Zinc finger CONSTANS
*Mi13g03290.1*	AATCGGAAGAATACATACAGC	CCTTAACACTACTCGAACCC	SOC1-like 2

### Metabolome Analysis

The analyses of the metabolome were executed as described previously (Chen et al., 2019). A detailed description of the methods is given below.

#### Sample Preparation and Extraction

The meristem (AF, BF, CF), flower bud (AS, BS, CS), Flowering phase 1 (AHF, BHF, BYF, CYF), and Flowering phase 2 (AHS, BHS, BYS, CYS) tissues of mango were freeze-dried (Scientz-100F), converted to a fine powder by crushing, dissolved 100-mg powdered sample in 1.2 mL of 70% methanol. Later on, we vortexed the samples for 30 s every half an hour, and this procedure was repeated six times followed by an overnight incubation at 4°C. On the subsequent day, the incubated samples were centrifuged for 10 min at 12,000 × g, filtrated through SCAA-104, 0.22 μm (ANPEL, Shanghai, China), and used for the UPLC-MS/MS analysis.

#### UPLC and ESI-Q TRAP-MS/MS Conditions and Bioinformatic Analyses

For metabolite analyses, we used a UPLC-ESI-MS/MS system (UPLC, SHIMADZU Nexera X2 and MS, Applied Biosystems 4500 Q TRAP) according to the following analytical conditions. For UPLC, Agilent SB-C18 (1.8 μm, 2.1 mm x 100 mm) columns were used. The two solvents that were used as the mobile phase, i.e., A and B included 0.1% formic acid together with ultrapure H_2_O and acetonitrile together with 0.1% formic acid, respectively. The gradient programming of the instrument to start the analysis was set to 95% and 5% of A and B, respectively, followed by a linear gradient of both A and B at 5% and 95%, respectively. This (mobile phase) composition was maintained for 1 min and was reversed (95% A and 5% B) for 1 min and 10 s. The same mobile phase conditions continued for additional 2 min and 55 s. The flow velocity, oven temperature, and injection volume were 0.3 mL/min, 40°C, and 4 μL, respectively. The remaining conditions for the compound detection and bioinformatic analyses were the same as those reported earlier by Sun et al. (2021). The VIP values were extracted from the OPLS-DA result, which was generated using the R package MetaboAnalystR; the data were log-transformed and mean-centered before OPLS-DA.

## Results

### Overview of Transcriptome Analysis

**Figure 2:**
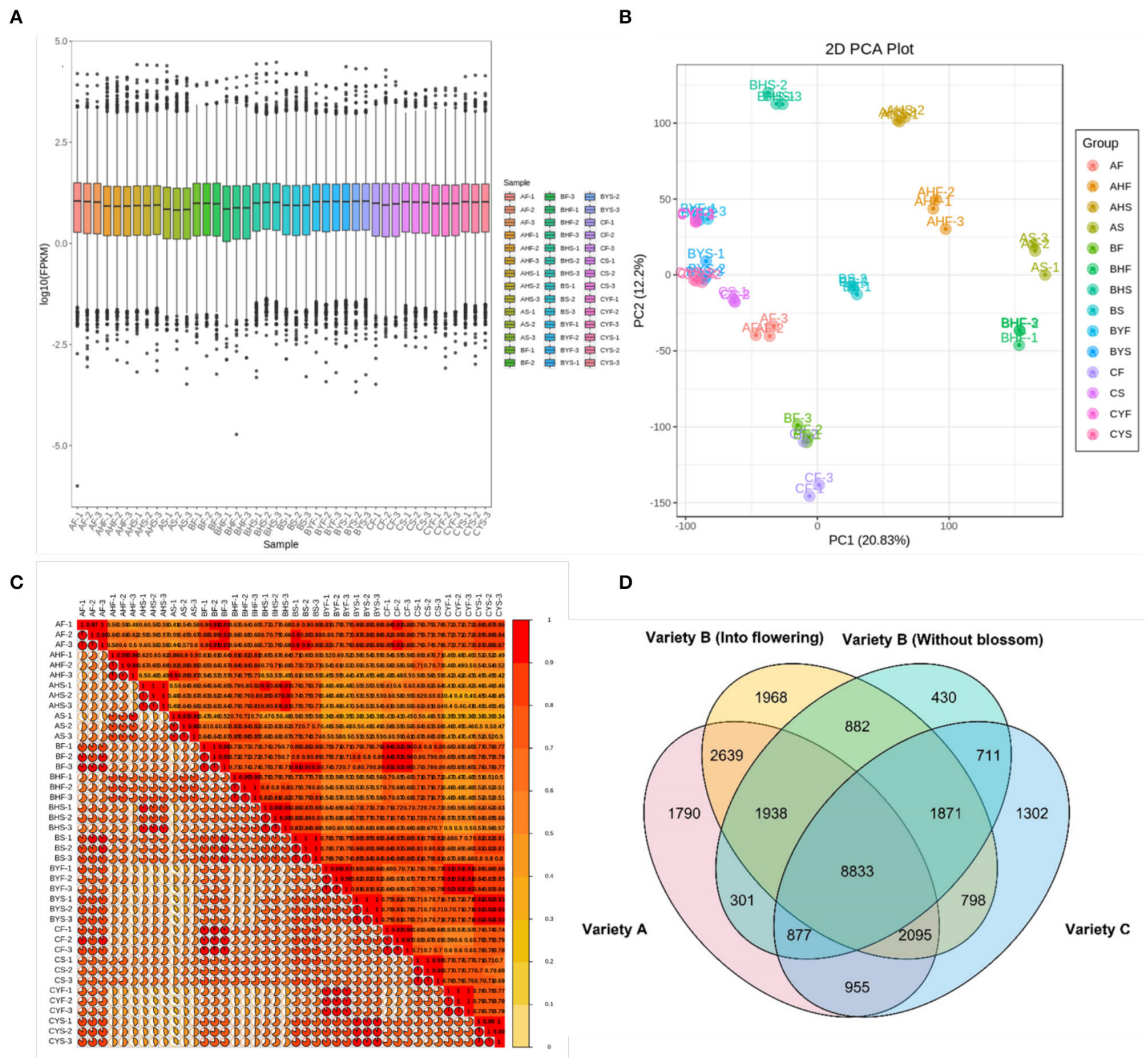
Summary of the transcriptome. **(A)** Gene expression levels as box plots of FPKMs. Ordinate represents Log10 FPKMs and Abscissa symbolizes sample names. The box plot for each region represents five statistics (from top to bottom: maximum, upper quartile, median, lower quartile, and minimum), the outlier is shown in black dots. **(B)** The PCA plot of all RNA-sequence samples. **(C)** The correlation heat map of the samples. **(D)** A Venn diagram representing common and specific DEGs in three mango varieties.

The project completed the transcriptome sequencing analysis of 42 samples, and ~301.95 Gb clean data were obtained. Each sample produced almost 6 Gb clean data, and the Q30 base percentage was 92% and above (Supplementary Table 1). After quality control, a comparison of clean reads was performed to the reference genome. It generated the position and unique sequence feature information on the reference genome for the sequenced sample. The transcriptome statistics are available in Supplementary Table 1. The proportion of generated sequencing reads that are successfully aligned to the genome is globally higher than 90%, which indicates that the reference genome is assembled good and the tested sequences are consistent with the reference genome and that there is no contamination in the experiment. A total of 36,242 annotated unigenes expression were detected and reported in terms of the FPKM values. As a comparable number of reads and coverage depths were generated among triplicates, an evaluation of FPKM box plots for gene expression levels of all genes in studied samples revealed that the sequencing results were dependable (Figure 2A). The PCA identified the two major components (PC1 and PC2) of the total variation, which represented 20.83 and 12.2%, respectively. The PCA analysis further validated our results and showed that easy-to-flower (variety A) and hard-to-flower (variety C) tissues fall away from each other (Figure 2B). Intermediate flowering (B variety) tissues fall in between both varieties. The correlation of gene expression levels (*R*^2^) was estimated. It acts as an essential indicator of experimental reliability and rationale of sample selection. When the value of the correlation coefficient is close to 1, it indicates that the expression patterns among samples possess a higher similarity. The *R*^2^ between biological replicate samples is >0.9 (Figure 2C; Supplementary Table 1).

### Differential Gene Expression Analyses

Gene expression quantification was used to identify the major transcriptional dynamics associated with the flowering and stage and/or variety-specific transcripts (Figure 2D). It was observed that 80, 92, 130, and 273 genes were specific to the AF, AS, AHF, and AHS stages, respectively. For variety B, a relatively higher number of genes were specific to BF (153 genes), BS (172 genes), BHF (262 genes), BHS (567 genes), BYF (230 genes), and BYS (288 genes) as compared to the same tissues of variety A. For variety C, 76, 135, 124, and 114 genes were specifically expressed during the CF, CS, CYF, and CYS stages, respectively. It was noticed that 8,833 genes were commonly expressed among variety A, variety B (into flowering and without blossom), and variety C (Figure 2D). Moreover, 1,790, 1,968, 430, and 1,302 genes were uniquely expressed in variety A, variety B (into flowering), variety B (without blossom), and variety C, respectively. For further analyses, we compared variety A and variety C since both had different phenotypes, i.e., easy-to-flower and hard-to-flower, respectively. Then, for confirmation of gene expression (trends), we used the transcriptome sequences of the tissues of variety B.

### Differential Expression of Flowering Pathways-Related Genes

**Figure 3:**
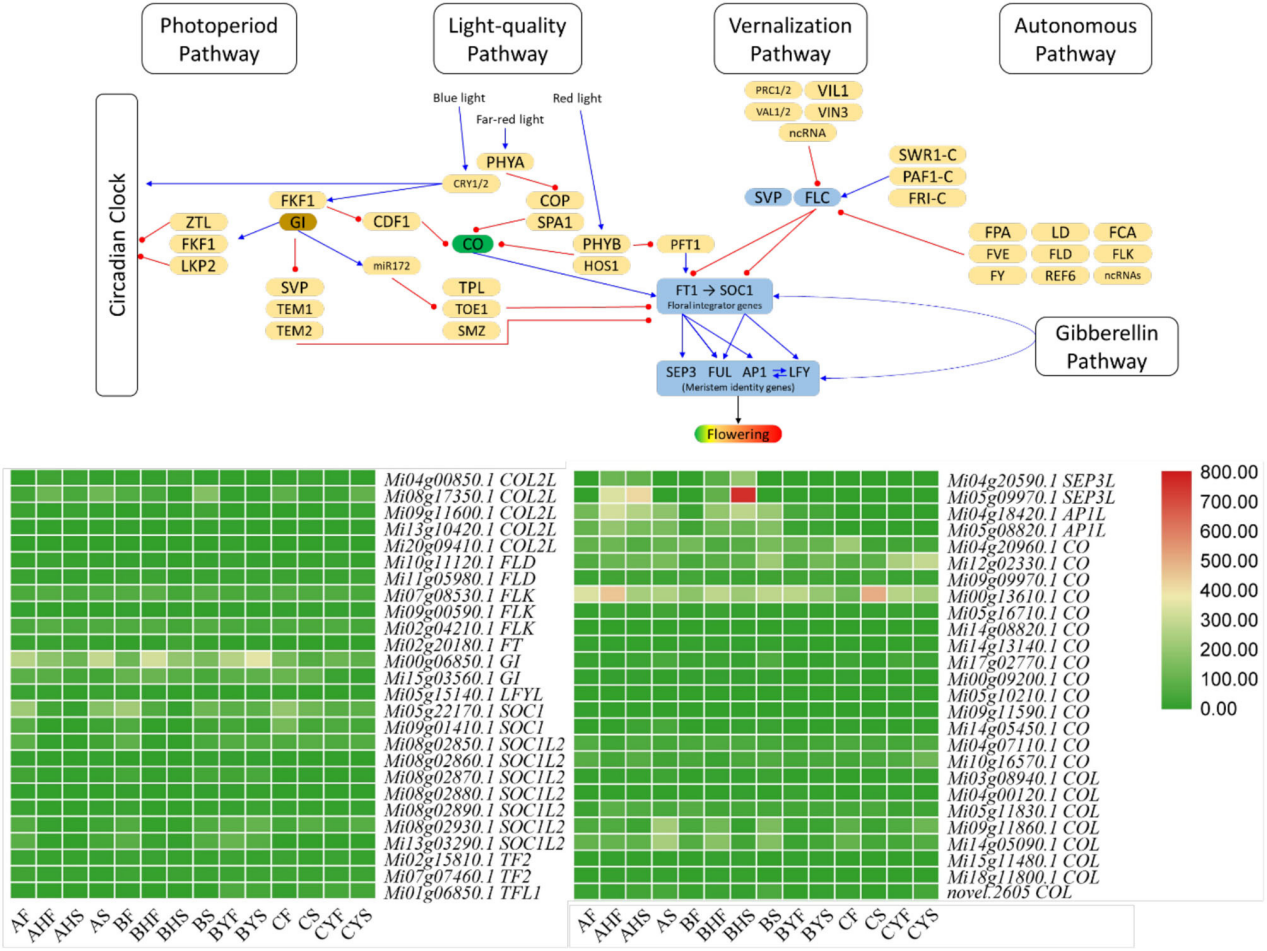
Flowering pathway in Mango. The heatmaps represent FPKM values of the major flowering-related genes, i.e., SEP3L (SEPALLATA3 like), APIL (AP1 like), CO (CONSTANS), COL (CONSTANS like), FLD (Flowering locus D), FT (Flowering locus T), LFYL (LEAFY like), GI (GIGANTEA), FLK (Flowering locus K), SOC (SUPRESSOR OF CONSTANS), SOCL (SOC like), TF (TERMINAL FLOWER), and TFL (TF like). The pathway figure was modified from the study of Kim (2020). The blue arrows show activation, while the red arrows show suppression. The expression of other genes in these pathways is presented in Supplementary Table 2.

Here, we studied four major flowering-related pathways, i.e., vernalization, gibberellin, photoperiod, and autonomous pathways. We studied expression variations of the genes that are associated with the four major flowering-related pathways. For this, we searched the KEGG and GO annotations of the DEGs from our transcriptome analysis and found 334 DEGs associated with these pathways. In these 334 DEGs, 105 were associated with 27 different genes (Figure 3; Supplementary Table 2). Then, 27 CO, 13 pseudo-response regulators (PPRs), 11 TOPLESS (and TOPLESS-related), 8 cyclic DOF factor (CDFs), and 5 EARLY FLOWERING 3 (ELF3) genes were differentially regulated between the 14 tissues of the three varieties.

#### Photoperiod Pathway's Core Components' Expression Is Consistent With the Flowering Potential of the Two Mango Varieties

We noted that the expression of FT was higher in all the tissues of variety A, i.e., AF, AHF, AHS, and AS, as compared to variety C, i.e., CF, CS, CYF, and CYS. However, the expression of FD (flowering locus D) that forms a dimer with FT was lower in CF and CS as compared to AF and AHF but higher in CYF and CYS as compared to AHS and AS. Further, the expression of the downstream genes such as that of SEP3L (SEPALLATA3-like; *Mi04g20590.1* and *Mi05g09970.1*) and AP1 (APETALLA1-like; *Mi04g18420.1* and *Mi05g08820.1*) was similar to FT, i.e., in variety A, their expression was higher, whereas their expression was very low (in most tissues, there was no expression) in variety C. Other than these, another meristem identity gene LEAFY-like (LFYL; *Mi05g15140.1*) was differentially expressed. A repressor of both AP1 and LFY, i.e., TERMINAL FLOWER (TF-L; *Mi01g06850.1*) had higher expression in variety C but near to zero FPKM values in variety A. Since FT is one of the core components of the photoperiod pathway in the flowering pathway, therefore, the higher expressions of FT, SEP3L, and AP2L in all tissues and of FD in AF and AHF in variety A as compared to variety C are consistent with the phenotype. Furthermore, the expression of the repressors, i.e., TF-L also supports their roles in the observed phenotype. Also, the higher expression of FT and SEP3L in B tissues except for BYF and BYS as compared to A tissues and the lower expression of TF-L in B tissues except for BYF and BYS as compared to A tissues support the above results. Moving further, we noted that the expression of several COs (*Mi18g11800.1, Mi05g11830.1, Mi03g08940.1*, and *Mi14g05450.1*) was higher expression in A as compared to C. Cos become attached to the proximal promoter of the FT; thus, it is understandable that the expression of COs is affecting FT and the downstream genes. The same expression trend was noted for the non-flowering and flowering tissues of B (Figure 3; Supplementary Table 3). However, the question remains, are there other genes/TF that are possibly regulating COs expression? We noted that CDF1, FKF1, and GIGANTEA (GI) were differentially regulated in the three studied varieties. Particularly, we found that the expression of a CDF1 (*Mi20g07310.1*) increased in C meristem (CF) as compared to A and B meristems. Two more CDF1s (*Mi02g05550.1* and *Mi07g07700.1*) had higher expression in BYF and CF as compared to AF. Our observations are consistent with the earlier known function of CDF1s that the expression of CO and FT in C is probably under the influence of CDF1 (Fornara et al., 2009; Goralogia et al., 2017). Our results showed that GI (*Mi00g06850.1* and *Mi15g03560.1*) and FKF1 genes had higher expression in all the tissues of variety A and variety B but lower in variety C. Because the Arabidopsis GI can directly bind to the FT promoter region and regulate its expression (Sawa and Kay, 2011), we can expect that the reduced expression of GI is directly influencing the lower FT expressions in variety C (Figure 3; Supplementary Table 2).

#### Light-Quality Pathway Is Less Likely to Contribute Toward the Flowering Phenotypes in A and C

The important players of light-quality pathway are phytochromes that are either activated or inactivated based on the incoming light. PHYs (PHYA and PHYB) together with SUPPRESSOR OF PHYA-105 (SPA) affect the accumulation of CO proteins [19]. CO accumulation is also controlled by the ubiquitin-ligase complex (CONSTITUTIVE PHOTOMORPHOGENIC 1 (COP1) and SPA1 are the part of it) [19]. The expression of the SPA1 genes was higher in AS, as compared to BS, BYS, and CYS. However, from the expression pattern, it is difficult to conclude any possible interaction between SPA1 and CO since it is not the expression of CO but its protein's accumulation that is affected by SPA1 (Ishikawa et al., 2006). We also detected the differential regulation of COP1 between the tissues of A, B, and C varieties. However, it did not open up a possible explanation of the changes in the expression of COs. Since PHYA and CRY1/2 stabilize CO protein (Kim et al., 2008), the higher expression of CRY1 in BS and CYS as compared to AS indicates that there is a possibility that CRY1 is stabilizing the CO protein in A that affects the FT expression, whereas, in case of variety C, this process is interrupted. Similarly, in the case of B, a slightly similar trend between CRY1 and FT expression can be related to the stability of CO in this variety, particularly, the plants which bear flowers. PHYB's differential but quite similar expression between the varieties and tissues indicates that destabilization of the CO protein by PHYB through ubiquination is not differentially happening in A, B, and C and that no delay in flower transitioning is underway specifically by this pathway (Valverde et al., 2004). Concomitantly, the expression of PFT1 (phytochrome and flowering 1 protein), which is present downstream of the PHYB, had a similar expression trend as that of PHYB in the three varieties. These observations indicate that it is less likely that the light-quality pathway might be affecting the CO and FT expressions in the mango varieties A, B, and C (Figure 3; Supplementary Table 2).

#### Flowering Repressors in the Vernalization Pathway Indicate Their Roles in the Flowering Phenotypes in A and C Varieties

Mango trees show distinct and different morphogenic responses to the changing temperature conditions (Kulkarni, 1988; Nunez-Elisea et al., 1996). Although we did not specifically establish this experiment for temperature effect on the flowering, we still were interested in the expression trends of the genes that are associated with the vernalization pathway. Two important flowering repressor genes, i.e., flowering locus C (FLC) and FRIGIDA (FRI; *Mi01g07210.1, Mi07g04370.1, Mi01g28170.1, Mi09g07130.1, Mi14g03970.1, Mi02g23420.1*, and *Mi02g23490.1*) (Henderson et al., 2003), showed lower expression in variety A and the flowering tissues of variety B as compared to C and the non-flowering tissues of variety B. This means that FLC should have directly inhibited the expression of FT and SUPPRESSOR OF CONSTANS 1 (SOC1) and affected flowering in variety C and the non-flowering type trees of B variety. We also noticed that VERNALIZATION INSENSTIVE 3 (VIN3) and VIN3-like (VIL) had higher expression in variety C and the non-flowering tissues of variety B (BYF and BYS). Another factor that plays a role in non-flowering or delayed flowering is SHORT VEGETABLE PHASE (SVP). There were five SVPs (*Mi01g16070.1, Mi04g06310.1, Mi15g07840.1, Mi18g07490.1*, and *Mi19g05730.1*) that had lower expression in the AS, AHF, BHF, and BHS stages as compared to respective tissues of variety C and the non-flowering type trees of variety B (BYF, BYS, CYF, and CYS). This indicates that SVPs are also active in B and C and cause the observed phenotype by the suppression of FT. Thus, these expression data indicate that FLC, FRI, and SVP play roles for the observed phenotype in mango variety C (Figure 3; Supplementary Table 2).

#### Gibberellin Biosynthesis and Signaling-Related Genes Are Likely Contributing Toward A and C Flowering Phenotypes

**Figure 4:**
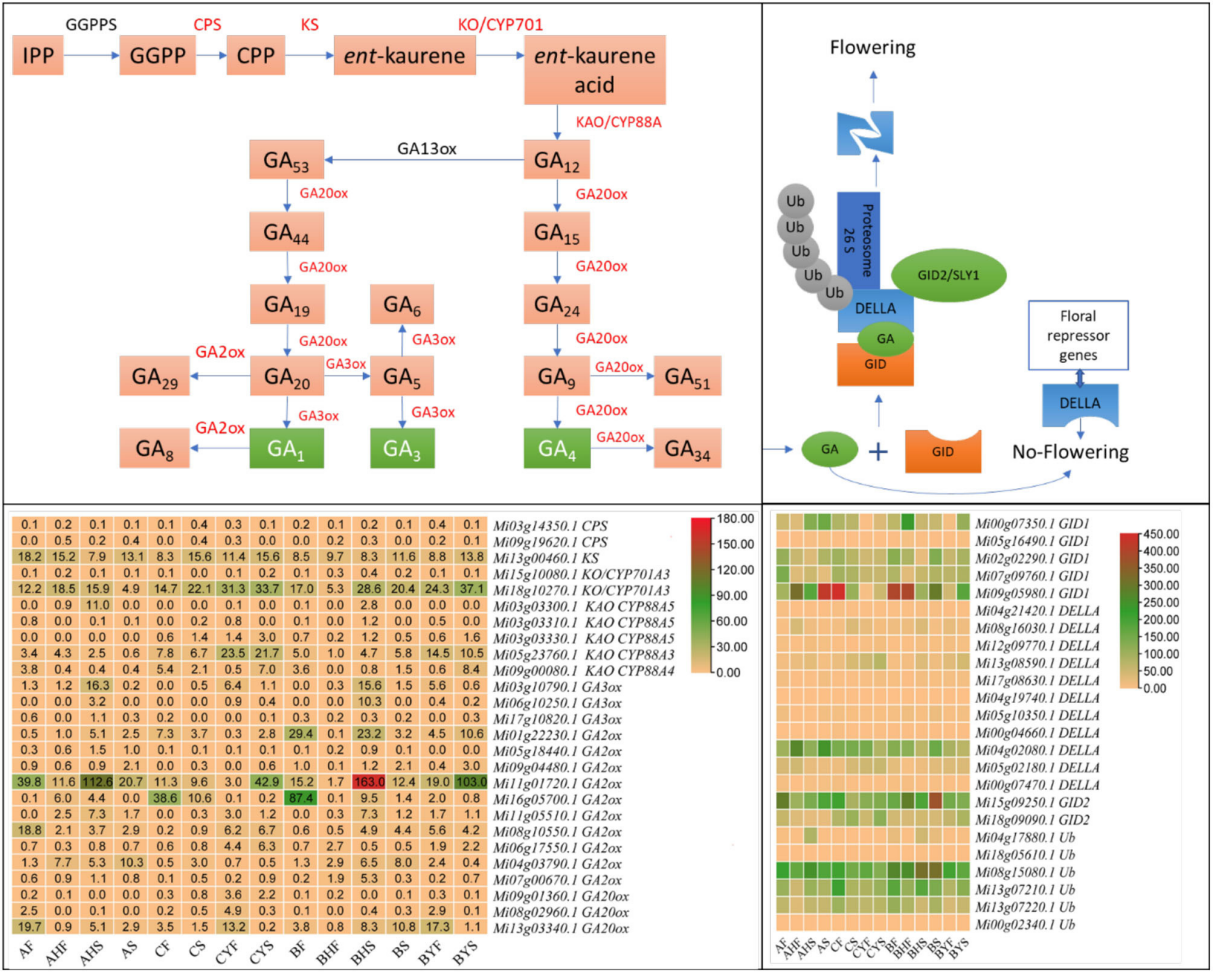
Gibberellin biosynthesis and signaling pathway in mango. The above panels show the key steps in GA biosynthesis and signaling. The respective heatmaps below the pathways show the FPKM values of related genes. Red genes were differentially expressed in GA biosynthesis. IPP, *iso*pentenyl diphosphate; GGPP, *trans*-geranylgeranyl diphosphate; CPP, *ent*-copalyl diphosphate; GGPPS, geranylgeranyl diphosphate synthase; CPS, *ent*-copalyl diphosphate synthase; KS, ent-kaurene synthase; KO, *ent*-kaurene oxidase; KAO, *ent*-kaurenoic acid oxidase; GA, gibberellic acid; ox, oxidase; GID, gibberellin receptor GID; DELLA, GRAS family protein; Ub, ubiquitin ligase. The blue broken box represents degraded DELLA protein.

The gibberellin (GA) is considered a key phytohormone for floral transition in plants (Tomer, 1984). GA homeostasis is attained by strict monitoring of activating and deactivating enzymes. We searched against GO and KEGG annotation using the keyword gibberellin and found 270 DEGs (Supplementary Table 3). Regarding the GA biosynthesis pathway, the expression of ent-copalyl diphosphate synthase (CPS) was very low in both varieties A and C. Whereas, we noted that ent-kaurene synthase (KS, *Mi13g00460.1*), ent-kaurene oxidase (KO, *Mi18g10270.1*), ent-kaurenoic acid hydroxylase (CYP88A3, *Mi05g23760.1* and CYP88A4, *Mi09g00080.1*), and gibberellin 3beta-dioxygenase (GA3-ox, *Mi06g10250.1*) had higher expression in C as compared to A tissues, indicating that GA biosynthesis is higher in C as compared to A. These changes imply that the ent-kaurene synthesis is higher in C tissues as compared to A tissues. This is based on their known roles in the biosynthesis of ent-kaurene (Zi et al., 2014; Lemke et al., 2019). Furthermore, the expression of CYP88A3/4 (Helliwell et al., 2001), GA3ox, and GA20ox indicates that, in C, the biosynthesis of biologically active GAs is going on. We observed that the expression of some of the GA2ox and GA20ox transcripts was lower in CF and CS but then increased in CYF and CYS as compared to their counterparts in A, indicating that, in C tissues, GA homeostasis is also active (Li et al., 2019). Among the regulators, we noted that some DELLAs (*Mi04g21420.1, Mi08g16030.1, Mi12g09770.1, Mi13g08590.1, Mi05g02180.1, Mi00g07470.1*, and *Mi17g08630.1*) showed increased expression in the C variety tissues but lower in the A variety tissues. Similarly, the expression of gibberellin receptor GID1 and F-box protein GID2 transcripts was higher in AF, AHF, AHS, and AS as compared to C tissues. Upon GA binding to its receptor GIDs, it undergoes a conformational change in the receptor. This change promotes its interaction with DELLAs. The expressions of the above genes were consistent in the flowering and non-flowering tissues of the B variety, thus confirming that these genes have functional roles in the observed phenotypes (Figure 4; Supplementary Table 3). Among TFs, we noted the differential expression of IBH1-like 1, MADS-box transcription enhancer factor, MADS-box TF, nuclear TF Y (gamma), MYB, LHY, MYC2, and WRKY33, WRKY22, bHLH. The expression of these TFs indicates that GA biosynthesis is also transcriptionally regulated (Supplementary Table 3).

### Differential Expression of Other Pathways

#### Auxin Biosynthesis and Signaling Are Variedly Active in Three Varieties

**Figure 5:**
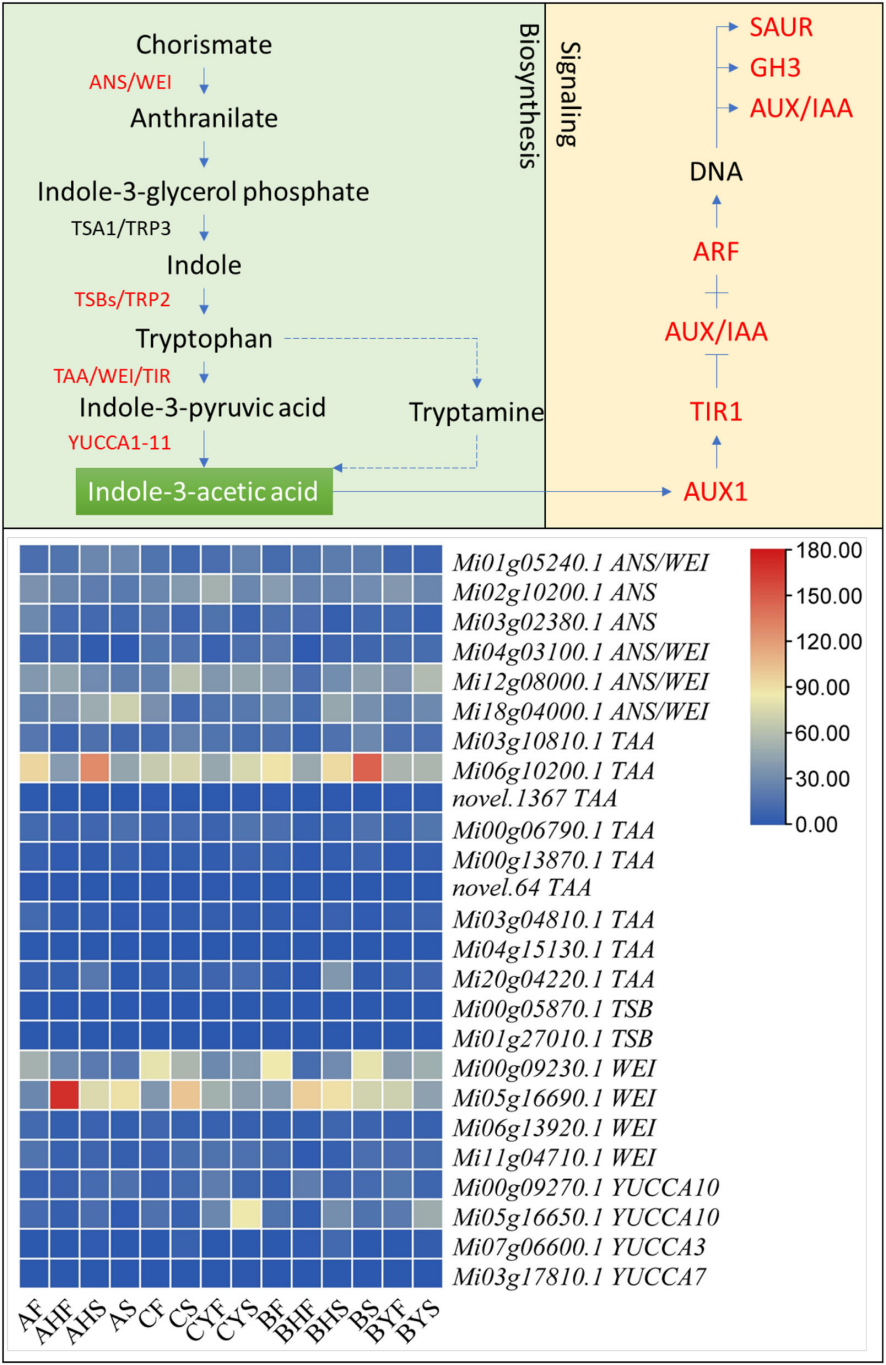
Indole-3-acetic acid biosynthesis and auxin signaling in mango. The red text shows differentially expressed genes. ANS, anthranilate synthase; WEI, weak ethylene insensitive; TSB. Tryptophan synthase beta chain; TAA, tryptophan aminotransferase; YUCCA, YUC flavin-containing monooxygenase; AUX1, auxin influx carrier; TIR1, F-box transport inhibitor response 1; Aux/IAA, auxin/indole-3-acetic acid; ARF, auxin response factor; SAUR, SAUR family protein; GH3, auxin-responsive GH3 family. The heatmap represents the FPKM values of the transcripts. The sample names are given as per Table 1.

We found 26 auxin biosynthesis-related genes that were differentially expressed between the three mango varieties and their studied tissues (Supplementary Table 3). Specifically, we noted the higher expression of anthranilate synthase (ANS) component I (*Mi01g05240.1*) and component II (*Mi18g04000.1*) in A tissues as compared to C. However, other ANS transcripts had the opposite expression pattern, i.e., higher in C as compared to A. Similarly, the weak ethylene insensitive proteins (WEI), tryptophan aminotransferases (TAAs), and YUC flavin-containing monooxygenases (YUCCAs) showed increased expression patterns in C as compared to A. A similar expression trend was noted for most of the above genes when we compared the flowering and non-flowering B tissues. We also noted that tryptophan synthase beta (TSB) chains were also expressed but their expression was very fractional. However, since tryptophan biosynthesis is an important step in IAA biosynthesis, it cannot be ignored. Overall, these changes indicate that indole 3-acetic acid biosynthesis in C tissues should be higher than that of A. Nevertheless, the higher expressions of some ANSs, WEI, TAAs, TSB, in A tissues indicate the possibility of higher biosynthesis of IAA (or its intermediates) (Figure 5; Supplementary Table 3).

Regarding auxin signaling, all the three core components, i.e., auxin influx carrier (AUX1), F-box transport inhibitor response 1 (TIR) auxin co-receptors, auxin/indole-3-acetic acid (Aux/IAA) transcriptional repressors, and the auxin response factor (ARF) TFs. Auxin promotes an interaction between TIF1 and Aux/IAA proteins, resulting in the degradation of Aux/IAA proteins and the release of ARF repression. The expression of AUX1s first decreased from AF to AS and then increased in AHF and AHS with slight differences. Whereas, in C, their expression increased from CF to CS and CYF but then decreased in CYS. Overall, the FPKM values in C were lower than those in A. In total, 37 IAAs, 7 TIR1s, and 33 ARFs were differentially expressed between the three mango varieties; their expression varied within and among the varieties, indicating a major auxin signaling interplay. This is evident from the expression of a large number of auxin-responsive genes, i.e., SAURs and GH3 transcripts (Figure 5; Supplementary Table 3).

#### Differential Regulation of Sugar Biosynthesis

In current study, there were five transcripts related to TPS1 in differentially expressed genes. Most of the TPS1 transcripts were highly expressed AS, AHF, and BHF as compared to the respective stages in difficult-to-flower tissues BYF, CS, and CYF (Supplementary Table 3). Similarly, several studies have indicated a strong involvement of sucrose transporters (SUC) in deciding flowering time (Cho et al., 2018). Transcripts related to SUC2, 3, and 4, sucrose synthase 1 (SUS1), SNF1-related protein kinase (KIN1), and NUTCRACKER-like proteins (NUC) were differentially regulated in all tissues, suggesting a complex involvement of these proteins in flowering (Supplementary Table 3).

#### Ambient Temperature Pathway-Related Genes Putatively Function in Mango Similar to Arabidopsis

Moderate changes in ambient temperature influence the transition to flowering (Sanchez-Bermejo et al., 2015). In the current study, four transcripts were identified to be related to *H2A.Z* (*Mi03g10660.1, Mi12g09820.1, Mi17g08680.1*, and *Mi06g10700.1*). These transcripts were highly expressed during all four stages of the three cultivars. However, the expression of PIF4-like transcript (*Mi04g00660.1*) was transiently upregulated in easy-to-flower tissues (AS and BHF) and very low to almost no expression in other tissues (Supplementary Table 3). The SHORT VEGETATIVE PHASE (SVP) is also central to thermoresponsive flowering and vernalization (Fernández et al., 2016). The five transcripts related to *SVP* (*Mi01g16070.1, Mi04g06310.1, Mi15g07840.1, Mi18g07490.1*, and *Mi19g05730.1*) were least expressed during the AS, AHF, BHF, and BHS stages as compared to respective difficult-to-flower tissues (BYF, BYS, CYF, and CYS). These findings clearly suggest potential functional conservation of these genes in Arabidopsis and mango for flower initiation.

### Quantitative Real-Time PCR Analysis

**Figure 6:**
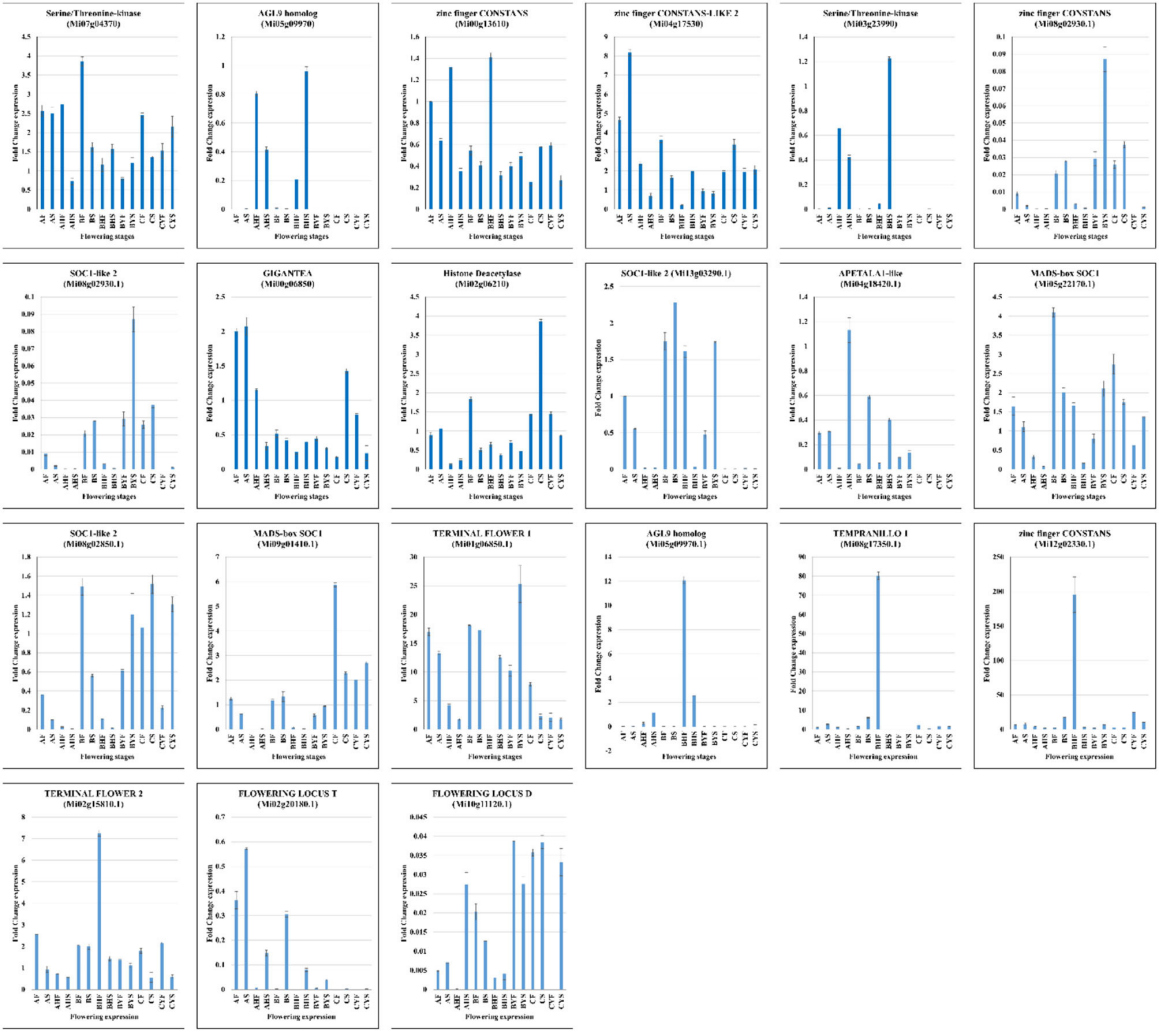
The RT-qPCR analysis of 21 mango genes from AF, AHF, BF, BHF, BYF, CF, and CYF. The *Actin* gene was used as the internal control. The error bars show the standard deviation.

The expression of 21 mango genes was confirmed through qRT-PCR analysis. The relative gene expression of the genes showed a similar trend as that of the FPKM values in the seven tissues belonging to three mango varieties (Figure 6). Furthermore, the expression changes in the flowering-related genes, i.e., GI, CO, FT, SOCs, FLD, and TEMPRANILLO 1, confirm our propositions regarding their roles in the differential flowering potential of the studied mango varieties.

### Comparative Metabolomic Profile of the Three Mango Varieties

**Figure 7:**
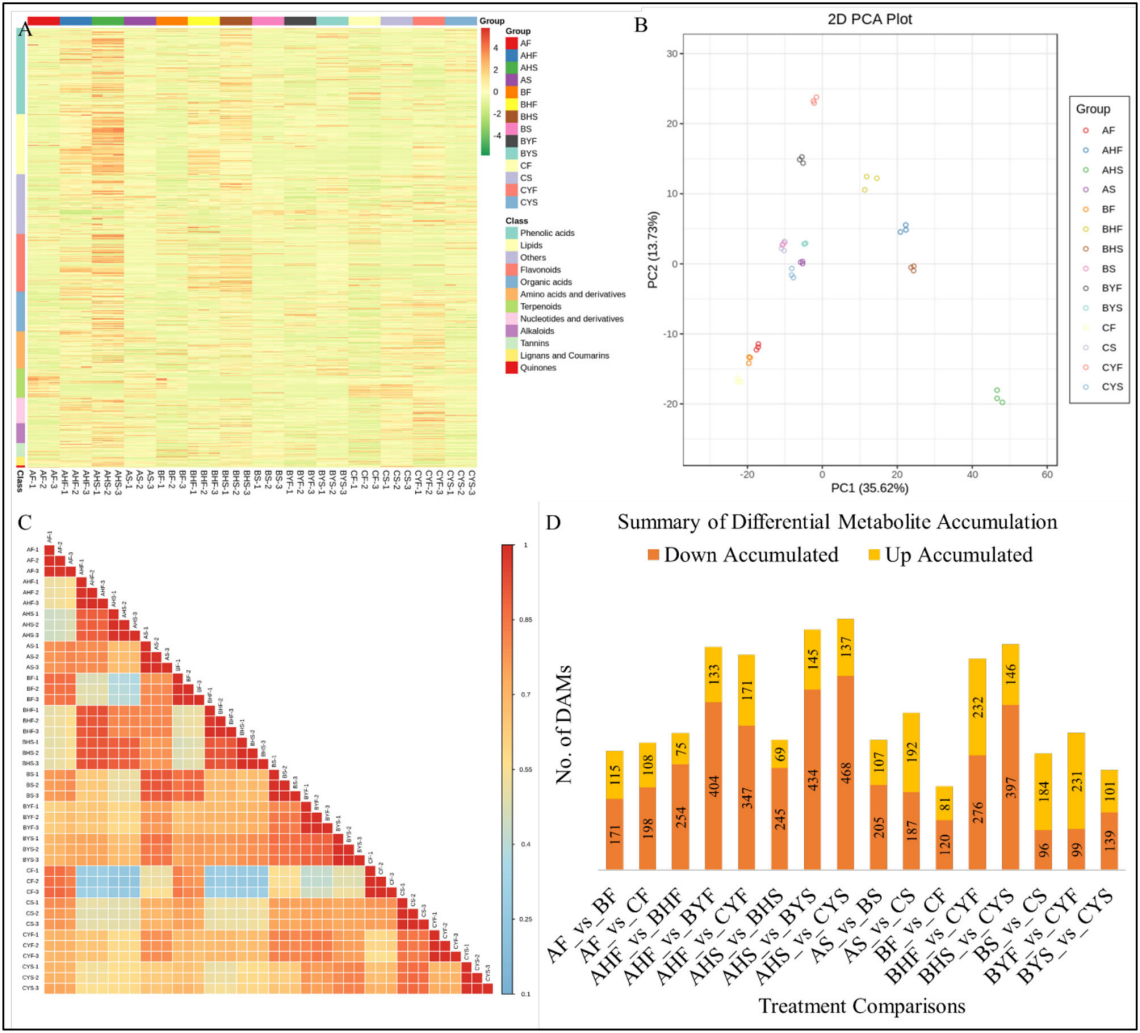
Summary of the metabolite profiling of mango tissues. **(A)** A heatmap of the relative content of the 1023 detected metabolites, **(B)** principal component analysis, **(C)** Pearson's correlation coefficient, **(D)** the number of upregulated or downregulated metabolites among the studied tissues of mango varieties A, B, and C, where A indicates Lippens), B indicates Banana Mango), and C indicates Linsen Mango). The tissue names are according to Table 1.

Metabolome profiling of 14 tissues belonging to three mango varieties differing in flowering resulted in the identification of a total of 1023 metabolites through UPLC-MS/MS (Figure 7A; Supplementary Table 4). The diversity of the detected metabolite could be confirmed from the broader classes of the detected metabolites, i.e., the metabolites could be classified as flavonoids, coumarins and lignans, tannins, terpenoids, quinones, phenolic acids, nucleosides and derivatives, lipids, amino acids and derivatives, alkaloids, organic acids, and others. The PCA analysis of the detected metabolites showed that the replicates of the same treatment grouped together, confirming the reliability of the sampling. The first and second principal components explained 35.62% and 13.73% variation, respectively (Figure 7B). We recorded a relatively higher PCC between AF, BF, and CF, AHF and BHF, AHS and BHS, and AS and BS. Whereas, the correlation between the C tissues (i.e., CS, CYF, and CYS) and B tissues (BYF and BYS) as compared with the A and B tissues was relatively lower, which is consistent with the observed phenotype (Figure 7C). The higher PCC indicates that the difference between the detected metabolites is more reliable (Inui et al., 2012).

### Differential Accumulation of Metabolites Correlates With the Transcriptome Profiles

The highest number of differentially accumulated metabolites (DAMs) was AHS vs. BYS and AHS vs. CYS (Figure 7D). The metabolites accumulated in the 14 different comparisons were enriched in 29 different KEGG pathways (Supplementary Figure 1). Most importantly, we observed that the DAMs were aminoacyl-tRNA biosynthesis, flavone and flavonol biosynthesis, linoleic acid metabolism, lysine degradation, pentose and glucuronate interconversions, phenylpropanoid biosynthesis, starch and sucrose metabolism, thiamine metabolism, and tryptophan metabolism between the flowering phase I and phase II in variety A as compared to leaf buds 3 and 4 of varieties B and C. These observations suggest that these pathways might be playing important roles in the observed phenotypes.

With regard to the observed changes in the expression of genes related to IAA, we specifically noted that anthranilate-1-O-sophoroside had higher content in C tissues (particularly CS, CYF, and CYS) as compared to A, indicating that the non-flowering type tissues had higher concentrations as compared to the flowering tissues. This was also noticed for the B variety tissues. We also noted that the concentration of indole-3-carboxaldehyde increased from AF to AHF and AHS but then reduced in AS. A similar accumulation trend was noted for C; however, the quantities in C were higher as compared to A. The concentration of methoxyindoleacetic acid and 1-Methoxy-indole-3-acetamide was also very much higher in C tissues as compared to A. These metabolites are enriched in the tryptophan metabolism pathway. The higher concentrations of other tryptophan and 5-hydroxy-L-tryptophan, N-acetyl-L-tryptophan, glycyl-tryptophan, and tryptamine also imply that C has higher concentrations of the key metabolites required for IAA biosynthesis. This is consistent with the transcriptome findings (Supplementary Table 4).

### Endogenous IAA and GA Levels Correspond to Their Respective Transcripts' Expressions

**Figure 8:**
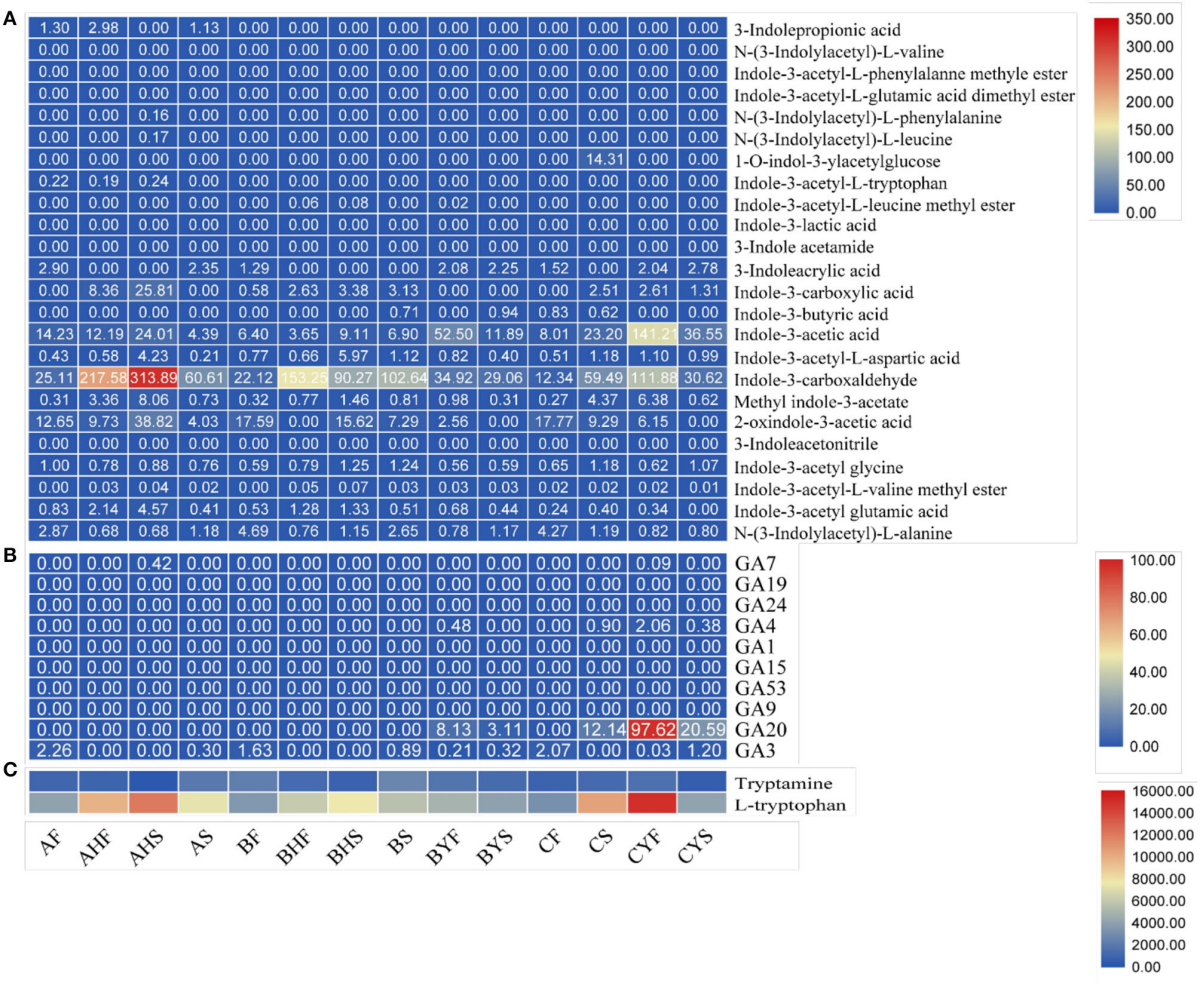
The relative content of auxins and GA in 14 mango flowering tissues quantified based on the ion intensity of different compounds. **(A)** auxins, **(B)** GA, and **(C)** amino acids (also classified as auxins in our analysis).

Since transcriptome analyses suggested that, auxin and GA biosynthesis and signaling are highly active in the studied tissues of the mango varieties, we measured the endogenous GA and auxin contents. The comparative analysis of the 14 tissues showed detection (accumulation) of 36 metabolites; 26 (including tryptamine and L-tryptophan) and 10 were classified as auxins and GAs, respectively (Figure 8). Interestingly, GA4 content was higher in variety C tissues and the non-flowering tissues of variety B, whereas the content of GA3 was quite similar in the three varieties (particularly, AF, BF, and CF). One interesting observation was the higher contents of GA3 (~4-fold) in CYF as compared to AS and BYS, which was ~1.34-fold higher than BS, indicating that variety C tissues have higher GA contents, which is consistent with the expression trends of GA biosynthesis-related genes. Whereas, GA20 was not detected in A and B flowering tissues but was detected in non-flowering B and C tissues, further confirming that GA regulation is active in the non-flowering tissues. Hence, these observations together with transcript expression changes and qRT-PCR analyses confirm that higher GA levels are related to the difficult-to-flower phenotype in mango (Figure 6).

The increased accumulation of IAA in all tissues of the three varieties confirms the observations in transcriptome and metabolome analysis (Supplementary Tables 3, 4), particularly very high accumulation of IAA in C. Because we noted significant changes in the expression profiles and accumulation of metabolites related to auxin biosynthesis, particularly IAA biosynthesis was higher in C as per expression profiles of ANS, WEI, TAA, YUCCAs, and TSBs. However, overall auxin accumulation was higher in A as compared to B and C variety tissues. In addition, the upstream metabolites, i.e., tryptamine and tryptophan, also showed varied accumulation patterns (Figure 8). This is also consistent with the observed changes in respective genes (Supplementary Table 3). From the expression changes and auxin accumulation, it can be concluded that higher auxin contents are related to the easy-to-flower phenotype.

## Discussion

### Interplay of FLOWERING LOCUS T and CONSTANS Together With Their Regulators/Repressors Modulate Flowering Robustness in the Studied Mango Varieties

Intensive molecular and genetic studies have shown that there are four major flowering pathways in plants, i.e., vernalization, gibberellin, photoperiod, and autonomous pathways. Apart from these, several studies have shown the involvement of several other pathways including ambient temperature-, hormone-, and sugar-dependent pathways (Kim, 2020). Among these, the photoperiod pathway's core components, i.e., CO and FT, are the major proteins for flowering in plants (Searle and Coupland, 2004; Borchert et al., 2005). The FT forms a dimer with FD (FT-FD dimer), which activates several SOC and several meristem activity genes (Turck et al., 2008). The expression of the FT and FD indicates that it must be a major cause of limited or no flowering in variety C. Other than these, the consistent expression pattern of SEP3Ls and AP1 in variety A and contrasting expressions in variety C strengthen the proposition that it is the FT expression that is limiting flowering in variety C but not in A and flowering tissues of B. These statements are based on the fact that SEP3 is required for meristem identity, leading to flowering (Hwan Lee et al., 2012), whereas the AP1 TFs are required for the transition from inflorescence meristem to floral meristem (Bowman et al., 1993). The lower expression of AP1 and other meristem identity genes like LFY coincides with the higher expression of TF genes. TF genes are repressors of LFY and API (Weigel and Meyerowitz, 1993) and in the C variety, and their reduced expression can be linked to higher expression of TF. Since CO attaches to the proximal promoter of FT; therefore, the higher expression of some COs in A and B tissues as compared to difficult to flower B and C can also be a reason for the similar expression of FT and resulting phenotypes. Furthermore, the increased expression of CDF1 in C meristem tissues and higher expression of GI and FKF1 in A and flowering tissues of B are relatable to the expression trends of CO and FT. This is based on the fact that CDF1 redundantly represses the transcription of CO and FT (Fornara et al., 2009; Goralogia et al., 2017) and that the GI and FKF1s repress CDF1s (Sawa and Kay, 2011; Song et al., 2012, 2014). Also, it is known that, in Arabidopsis, GI can directly bind to the FT promoter region and regulate its expression (Sawa and Kay, 2011); thus, we can expect (based on the expression patterns of FT and GI) that the reduced expression of GI is directly influencing the lower FT expressions in variety C. Thus, it is the expression changes in FT and CO together with their activators/repressors that are governing flowering robustness in the studied mango varieties.

Although we ruled out the possible role(s) of the light-quality pathway-related genes, the roles of vernalization pathway-related transcripts are relatable to the phenotypes of the three mango varieties. Particularly, the expression trends of FLC and FRI indicate inhibition of FT and SOC1 in variety C and non-flowering variety B. This is consistent with the known repressor roles of FLC and FRI in Arabidopsis and the fact that FRI upregulates the expression of floral repressor FLC (Henderson et al., 2003). The lower expression of SVPs in A and B flowering tissues as compared to that of difficult-to-flower B and C tissues indicates flowering suppression, probably by suppressing FT expression. SVPs play roles in delayed or no-flowering by forming a complex with FLC to repress the expression of genes that initiate flowering (Mateos et al., 2015). Thus, we can conclude that flowering repressors such as FLC and FRI are related to the mango flowering robustness (Figure 8).

### Higher GA Acid Biosynthesis and Signaling Are Associated With the Difficult-to-Flower Mango Phenotype

One of the key phytohormones in floral transition in plants is GA (Tomer, 1984). In mango, it has long been established that GA inhibits flowering (Tomer, 1984). Similar repressing reports are known for woody perennials, e.g., apple (Zhang et al., 2019). The study in apple reported the regulation of gene expression related to two major pathways, i.e., diterpenoid biosynthesis and plant hormone signal transduction. This is consistent with our findings too. The higher synthesis of GAs in variety C tissues can directly be linked to respective higher expression of CPS, KS, KO, and KAO (Zi et al., 2014; Lemke et al., 2019). The reduced GA20ox and GA30x expression in easy-to-flower tissues of varieties A and B as well as their expression trends in variety C indicate adjustment of GA biosynthesis or the feedback and feed forward mechanism of GA regulation. These mechanisms are essential for a plant to maintain transcriptional responsiveness and adjust growth and development (Thomas and Sun, 2004). The higher expression of gibberellin receptor GID1 and F-box protein GID2 in easy-to-flower tissues is consistent with the observations in apple. These observations indicate that GA contents are inversely related to GID expression. Also, it might indicate that GA homeostasis may also be regulated by the modulation of GA receptors (GIDs) apart from GA metabolic genes, as witnessed in Arabidopsis (Griffiths et al., 2006). Our observation of the varied expression of the different transcripts of DELLA reflects GA regulation. Also, the higher expression of multiple DELLAs in C (particularly in CS and CYF) is consistent with that of FLC. The interaction of both DELLA and FLC has been previously studied in Arabidopsis, where it was reported that both play negative roles in flowering and that their interaction enhances the transcriptional repression ability of FLC (Li et al., 2016). Furthermore, the expression trends of DELLAs together with SOC1 and LFY genes strengthen our consideration of direct repression of flowering by GA in mango (C and difficult to flower B variety). This is because both SOC1 and LFY are downstream targets of DELLAs in Arabidopsis (Moon et al., 2003; Zhang et al., 2019). Overall, our results indicate that variation in the expression of GA metabolic and signaling pathways helps mango varieties to achieve GA homeostasis and that the higher GA levels are associated with the difficult-to-flower phenotype or *vice versa*. Finally, our findings that TFs that showed up in our GO annotation search against GA give potential candidates for mediating GA-driven flowering robustness.

### Higher Auxin Levels Are Associated With the Easy-to-Flower Phenotype

Auxin regulates floral meristem initiation as well as other aspects such as flower initiation, its growth, and the reproductive success of the flower (Sundberg and Østergaard, 2009). Somewhat variable expression of the auxin biosynthetic genes in the studied mango tissues indicates that, with the floral transition, several tissues may exhibit unique/different auxin levels. Since the tissues of the easy-to-flower varieties accumulated higher quantities of auxins (Figure 7), that most of the ARFs had higher expression in these tissues, which shows a direct link with the LFY expression (Supplementary Tables 2 and 3). We say this because an earlier report on the molecular framework of auxin-mediated flowering indicated that auxin (and resultantly the expression of ARF) triggers the initiation of flower primordium by increasing LFY expression (Yamaguchi et al., 2013). At the same time, the dormancy-associated protein 1 (DRM1, *Mi06g15100.1*) followed an increasing and then a decreasing trend in easy-to-flower tissues and followed an opposite trend in hard-to-flower tissues. This gene is downregulated by high levels of auxins (Rae et al., 2014). This suggests a possible effect of auxins on breaking bud dormancy. A link between auxin and flower development was first established when the auxin transport mutant *pin1* (Auxin efflux carrier) was isolated and characterized [for references please see (Cheng and Zhao, 2007)]. The inflorescence of *pin1* often does not have any flowers. In the current study, there were 10 transcripts related to auxin efflux carrier family proteins (KEGG entry K13947). Four of these were homologs of *PIN1* (*Mi08g15630.1, Mi13g07790.1, Mi14g15240.1*, and *Mi20g11470.1*). Interestingly, most of these transcripts were downregulated from the AF to AHS stages and followed an opposite trend in difficult-to-flower tissues (from CF to CYS). This downregulation is potentially related to a decrease in auxin polar transport, which apparently fails to stop the transition from vegetative growth to reproductive growth. The conversion of an inflorescence meristem to a flower meristem apparently requires normal polar auxin transport (Cheng and Zhao, 2007).

### Other Possible Mechanisms Related to Flowering Robustness in Studied Mango Varieties

Our results indicate that there could be other mechanisms that could be associated with the observed differences in mango flowering robustness in A, B, and C varieties. One of the two mechanisms is changes in sugar biosynthesis. In transcriptome analyses, we observed higher expression of TPS1 transcripts in easy-to-flower A and B tissues, which indicate higher trehalose-6-phosphate biosynthesis. T6P acts as a flowering induction signal in Arabidopsis. Sucrose seems to function primarily in the leaf phloem to enhance the generation of florigens such as FT, while T6P functions in the shoot apical meristem to promote the flowering signal pathway downstream of those florigens. In Arabidopsis plants where TPS1 is downregulated, the expression of FT and TWIN SISTER OF FT (TSF) is delayed while that of its upstream regulators, such as CO and GI, are not affected (Wahl et al., 2013). Overexpression of TPS1 causes early flowering under both LD and SD conditions (Supplementary Table 3). Since the metabolite data confirmed higher accumulation of both T6P and sucrose in easy-to-flower tissues, we can link the TPS1 expression with T6P levels and resultant FT generation based on the above-mentioned reports.

The second mechanism involves changes in transcripts related to the ambient temperature pathway, which can also be associated with the changes in the expression of FT and the observed variations in flowering robustness in A, B, and C mango varieties. It is essential to note that thermoresponsive flowering is less understood than vernalization because multiple independent thermoresponsive pathways are involved (Capovilla et al., 2015). The histone variant H2A.Z acts as a thermosensor for flowering time. The occupancy of H2A.Z at the FT promoter is negatively affected at increased temperatures. H2A.Z facilitates the binding of PHYTOCHROME INTERACTING FACTOR 4 (PIF4) to the FT promoter (Kumar and Wigge, 2010; Kumar et al., 2012) to activate the FT expression in cooperation with CO under warm ambient temperatures (Kumar et al., 2012). The higher expression of *H2A.Z* during all the four stages of the three cultivars cannot explain the FT expression. However, the transient upregulation of PIF4 in easy-to-flower tissues and contrasting FPKM values in other tissues can be linked to FT expression and resulting phenotypes. Similarly, the expression of flowering repressor SVP's transcripts, which is also central to thermoresponsive flowering and vernalization (Fernández et al., 2016), indicates the repression of flowering in C but not in A. These findings clearly suggest potential functional conservation of these genes in mango for flowering robustness.

## Conclusion

This study investigated the transcriptome and metabolome profiles of flower-related tissues in three mango varieties differing in their flowering intensity (easy-to-flower, intermediate flowering, and hard-to-flower). A significant variation was observed in the number of transcripts expressed in a stage and/or variety-specific manner. The expression of transcripts related to the major flowering pathways, i.e., vernalization pathway, photoperiod pathway, light-quality, and autonomous pathway, indicated the importance of major genes such as FT, SEP3L, APIL, CO, GI, LFYL, SOC, SOCL, TF, SPA1, FRI, FLC, and SVP. The comparative transcriptome and metabolome data supported with qRT-PCR and endogenous hormone levels indicate that a variety of both activators and repressors are interplaying and affecting the major flowering-related and meristem identity-related genes. Our data showed that GA and auxin biosynthesis and signaling have important roles in flowering robustness in mango. Together with the flowering-related pathways, hormone biosynthesis and signaling (GA and auxin), sugar biosynthesis, and ambient temperature pathways are modulating the observed phenotypes.

## Data Availability Statement

The datasets presented in this study can be found in online repositories. The names of the repository/repositories and accession number(s) can be found in the article/Supplementary Material.

## Author Contributions

QL and SW: conceptualization. RZ: methodology and data curation. KS: software and visualization. KS, ML, and JY: validation. JW: formal analysis. ML: investigation. JC: resources and supervision. QL: writing—original draft preparation, project administration, and funding acquisition. SW: writing—review and editing. All authors contributed to the article and approved the submitted version.

## Funding

This research was funded by the National Key R&D Program of China (2019YFD1000500); the Natural Science Foundation of Hainan Province (2019CXTD410); the Natural Science Foundation of China (31471849 and 31901968); Accurate identification of mango germplasm resources of Ministry of Agriculture and Rural Affairs (19211144); and the Conservation Fee Project of Species Resources in Ministry of Agriculture and Rural Affairs (125163006000160001).

## Conflict of Interest

The authors declare that the research was conducted in the absence of any commercial or financial relationships that could be construed as a potential conflict of interest.

## Publisher's Note

All claims expressed in this article are solely those of the authors and do not necessarily represent those of their affiliated organizations, or those of the publisher, the editors and the reviewers. Any product that may be evaluated in this article, or claim that may be made by its manufacturer, is not guaranteed or endorsed by the publisher.

## Supplementary Material

Supplementary Figure 1 The first panel shows the summary of pathways to which DAMs were enriched in mango tissues. Red, significantly enriched; Blue, enriched; Sky blue, not enriched. The x-axis shows the treatment comparisons, and the y-axis shows the KEGG pathway names. The second panel shows a heatmap of the DAMs enriched in seven important KEGG pathways that were differentially regulated between the three mango varieties. The tissue names are according to Table 1.

Supplementary Table 1 Summary of the statistics of transcriptome sequencing of 14 mango tissues of varieties A, B, and C.

Supplementary Table 2 FPKM values of the DEGs associated with four flowering-related pathways in mango varieties A, B, and C differing in flowering intensity.

Supplementary Table 3 FPKM values of the DEGs enriched in important pathways associated with flowering in three mango varieties differing in flowering intensity.

Supplementary Table 4 The list of differentially accumulated metabolites and their respective intensities in the studied tissues of the three mango varieties.

## Abbreviations

AP1: *APETALA 1*
*RING1A*: *ARABIDOPSIS THALIANA RING 1A*
*CO*: *CONSTANS*
COP1: CONSTITUTIVE PHOTOMORPHOGENIC 1
CRY1/2: Cryptochrome Circadian Regulator 1/2
CDFs: *cyclic DOF factor*
ELF3: *EARLY FLOWERING 3*
FKF1: Flavin-binding, kelch repeat, f box 1
FT: *FLOWERING LOCUS T*
FTL: FLOWERING LOCUS T-like
FPKM: Fragments Per Kilobase of transcript per Million fragments mapped
FRI: FRIGIDA
FLC: Flowering locus C
GWAS: Genome-wide association studies
GA: Gibberellin/gibberellic acid
Hd3a: Heading date 3a
JAOMT: Jasmonate O-methyltransferases
LFY: LEAFY
NUC: NUTCRACKER-like proteins
PIF4: PHYTOCHROME INTERACTING FACTOR 4
PPRs: Pseudo-response regulators
QTLs: Quantitative trait loci
SEP3: SEPALLATA3
SEP3L: SEP3 like
SVP: *SHORT VEGETABLE PHASE*
KIN1: SNF1-Related Protein Kinase
SUS1: Sucrose synthase 1
SUC: Sucrose transporters
SPA: SUPPRESSOR OF PHYA-105
TF: TERMINAL FLOWER
TPL: TOPLESS
TPR: TOPLESS-related
TPS1: TREHALOSE-6-PHOSPHATE SYNTHASE 1
TSF: TWIN SISTER OF FT
VIN3: VERNALIZATION INSENSTIVE 3
VIL: VIN3-like.
