# Supplementary figures and images for: Transcriptome and Metabolome Analyses Reveal the Involvement of Multiple Pathways in Flowering Intensity in Mango
Source: Front Plant Sci. 2022 Jul 14;13:933923. doi: 10.3389/fpls.2022.933923 (PMC9330041; doi:10.3389/fpls.2022.933923)

## Slide 1
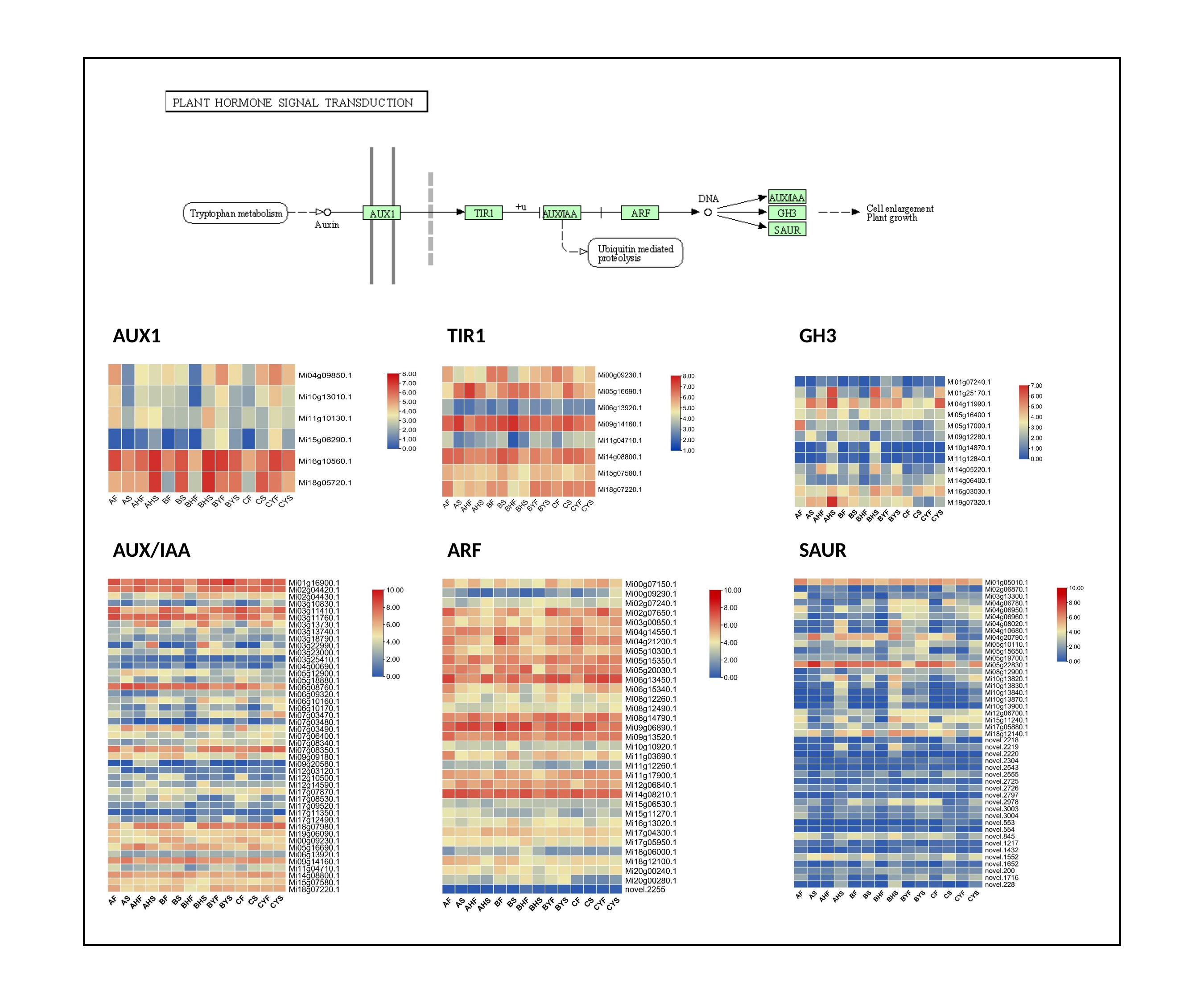

AUX1
TIR1
GH3
AUX/IAA
ARF
SAUR

Supplement: Supplementary Figure 1 — The first panel shows the summary of pathways to which DAMs were enriched in mango tissues. Red, significantly enriched; Blue, enriched; Sky blue, not enriched. The x-axis shows the treatment comparisons, and the y-axis shows the KEGG pathway names. The second panel shows a heatmap of the DAMs enriched in seven important KEGG pathways that were differentially regulated between the three mango varieties. The tissue names are according to Table 1. [file Presentation_1.PPTX]
